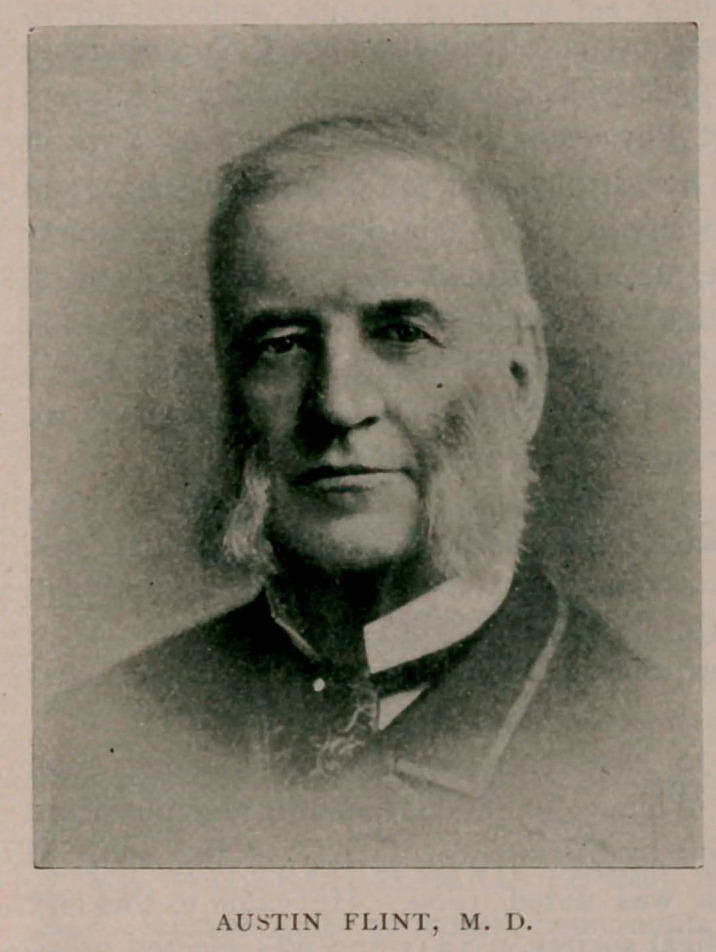# A Century of Medical History in the County of Erie—1800–1900

**Published:** 1898-05

**Authors:** William Warren Potter

**Affiliations:** Buffalo, N. Y.


### A CENTURY OF MEDICAL HISTORY IN THE COUNTY OF ERIE—1800-1900.

BY WILLIAM WARREN POTTER, M. D., Buffalo, N. Y.

*Pioneer Physicians—Medical Societies—Medical Colleges—Hospitals—Medical Journals—Medical Officers of the Civil War—Women Physicians—History of Homeopathy—Individual Members of the Profession.*

[*Continued from the April edition.*]

AT THE end of the first decade, that is, in 1831, the annals of the society indicate that very little progress had been made by the medical profession during that period. It does not appear to have improved in *esprit de corps* to any appreciable degree, nor does its *personnel* seem to have bettered itself in quality or quantity. The names of twenty or more members appear but once on the records and only a total of twenty are found on the roll in 1831. Of the original charter members but nine remained—namely, Cyrenius Chapin, Josiah Trowbridge, John E. Marshall, Benjamin C. Congdon, of Buffalo, Charles Pringle, of Hamburg, Rufus Smith and Jonathan Hoyt, of Aurora, Ira G. Watson, of Wales, and William H. Pratt, of Eden. The following names were recorded on the secretary's book, but they do not appear to have completed their membership—namely, Daniel Allen, Nathaniel R. Olmstead, Isaac Dunning, John Allen, Henry Hitchcock, Thaddeus Hubbard, Parley B. Spaulding, James M. Smith, Jonathan Foote, Daniel H. Orcott, Israel Congdon, Alvin Cowles, Sidney R. Morris, Marvin Webster, John D. Fisk, Edward J. Durkee and W. P. Proudfoot.

If the profession of medicine and the medical society did not make substantial advance during the period from 1821 to 1831, it must be admitted in extenuation that this was a period of privation, embarrassment and distress. The people were for the most part poor, or at least not wealthy. They had hardly recovered from the effects of the war and there was but little capital with which to carry on great enterprises. If, then, the people themselves were not prosperous, how could the medical profession expect to advance? It was a time, too, when quackery was gaining foothold; red pepper and lobelia represented advanced therapy, while Thompsonian and steam doctors were abroad in the land. The conditions, in short, were those generally pertaining to newly settled regions. Under such environment it may be easily understood that the practice of medicine was carried on by a few faithful followers of the science at a disadvantage that was well-nigh discouraging, and which would have dismayed hearts less stout than theirs. Some, indeed, were obliged to supplement their already scanty incomes by engaging in other pursuits part of the time, while others felt compelled to abandon the profession altogether.

The medical society keenly felt the effect of the hard times and in 1825 sought to replenish its treasury by raising its fee for membership from $2 to $5. The annual dues remained at $1, as before. That an expedient of such doubtful propriety should fail of its purpose is not surprising. Only two new members joined during the succeeding three years.

The society, however, was not idle during this period. It appears to have been among the first in the state to appreciate the value of vital statistics, for a bill on this subject was drafted under its direction, which was sent to the legislature for action. It also devoted much attention to the subject of vaccination and was always aggressive in the various branches of medical science. A number of men, too, joined the society during this period who deserve special notice.

Dr. Bela H. Colgrove, of Sardinia, was one of these. He was a native of Rhode Island, a graduate of the College of Physicians and Surgeons of New York and settled in Sardinia in 1820. He joined the society in 1823 and was president in 1828. He resided in Buffalo for a few years and was associated in practice with Drs. Trowbridge and Marshall. Finally, he returned to Sardinia, where he continued his professional work for about fifty years. He became famous as a surgeon and his services were in demand in adjoining counties. He died March 19, 1874, aged 77 years.

Moses Bristol was born in Oneida county, came to Buffalo in 1822 and joined the society in 1823. He held the office of censor during the years 1834, 1836, 1837, 1839 and 1840, was president in 1833 and again in 1838. He continued in active practice until 1849, when his health began to fail, but he lived until 1869. Dr. Bristol did much to maintain the character and standing of the profession during the period of his activity. Of others who joined the society in 1823, we find the names of Orlando Wakelee, of Clarence, and

Emmons S. Gould. Benjamin C. Congdon, one of the founders, was treasurer of the society for one year, from 1823 to 1824, and continued as a member until 1833.

Henry Rutger Stagg became a member of the society in 1824. He was secretary and librarian in 1828, again secretary in 1833 and president in 1834. He was a man of attainments, possessed a literary taste, but withdrew from the ranks of the profession, severing his connection from the society in 1836. He became associate editor of the *Buffalo Journal*, a weekly newspaper, in which occupation he continued until 1838. Of the other members who joined the society in 1824 we may mention Erastus Wallis, of Aurora, Judah Bliss, of Buffalo, and Carlos Emmons, of Springville. In 1826 but two additional names are recorded—namely, Michael Martin and Stephen Dean, the latter locating at East Hamburg. Ira Shedd, a licentiate of the society, appears on the rolls during 1827 ; likewise Orson Cary, the latter becoming a censor in 1830.

Carlos Emmons established himself at Springville, joined the society in 1824, was elected vice-president in 1833, president in 1834, and a delegate to the state society in 1841. He also served as a member of the assembly and in the senate.

Erastus Wallis, of Aurora, became a member in 1824, vice-president in 1839, president in 1840, and served several years as a censor. He came to Buffalo in 1853, where he resided a few years and then returned to Aurora. He was a member during thirty-eight years and died in 1862. In 1828 J. S. Trimble joined the society ; John M. Harrington, a licentiate, became a member in 1830, as also did Orson S. St. John and Lucien W. Caryl. D. J. Williams, of Aurora, joined in 1831.

We have already shown why the medical society failed to grow in numbers during the first decade of its existence, but now a new and prosperous epoch seemed dawning. Buffalo more than trebled its population in the five years between 1825 and 1830, while the county of Erie, exclusive of Buffalo, more than doubled its inhabitants during the same period of time.

Bryant Burwell, a native of Herkimer county, came to Buffalo in 1824 and joined the society in 1825. He was associated in practice with Dr. Cyrenius Chapin for some years. He was vice-president in 1832 and a delegate to the state society in 1833. He was appointed by the state society one of a committee of three to examine the medical laws of the state with reference to any amendments needed ; also he was made a member of a committee to obtain an opinion from the attorney-general upon the question of the powers of medical societies as to the admission of members, which was an important question then and has always been one of moment. He became a permanent member of the state medical society in 1837. Dr. Burwell represented the Buffalo Medical Association at the initial convention held in New York, in 1846, with reference to organising a national medical society and he was a delegate to the first and second meetings of the American Medical Association, held respectively at Philadelphia in 1847 and at Baltimore in 1848. Again, in 1850, he represented the city association at the Cincinnati meeting of the American Medical Association. He was a censor of the state medical society in 1847, 1848 and 1850, and a member of the committee of correspondence of that society for several years. Dr. Burwell maintained an active part in the deliberations of the county society until 1854 and was one of its censors for many years. He died September 8, 1861, aged sixty-five years, having maintained the respect and confidence of his professional confrères during all the years of his residence in Buffalo.

Alden S. Sprague, another strong character, a native of New Hampshire, came to Buffalo a year later than Dr. Burwell,—namely, in 1825, and was elected a member of the county society in 1826. He was treasurer from 1829 to 1833 inclusive, and was chosen president in 1835, during which year he was also health physician of Buffalo. In 1851 he was again elected president, but ceased to be an active member in 1852. He was a delegate to the state society in 1839 and again in 1845, and was elected a permanent member of that body in 1847. He died January 7, 1863, but little more than a year after Dr. Burwell, with whom he had been a contemporary for thirty-seven years. Dr. Sprague was recognised as one of the foremost physicians of his period, and he obtained also a deserved fame as a surgeon.

Harry H. Bissell, a native of Vermont, came to Clarence in 1828, during which year he joined the society. Afterward he removed to Lancaster, where he was associated in practice with Dr. Hyde. Finally, he returned to Buffalo, was elected president of the society in 1836 and also served as a censor for many years. He was sent as a delegate to the state society in 1837.

Luther Spaulding, of Williamsville, became a member of the society in 1831, although he had been a resident of the county since 1821. In 1832, Alden Thomas, Arba Richards, of Wales, Horace B. Camp, of Aurora, and Josiah Barnes, Joseph R. Jones and James Edwin Hawley, of Buffalo, became members of the society.

Charles Winne, a native of Albany, came to Buffalo in 1833, in which year he also joined the society. He was chosen a delegate to the state society in 1834 and was health physician of Buffalo in 1836. He served as treasurer of the society during the years 1836, 1837 and 1838; was secretary in 1845–46, and was associated in practice for some years with Dr. Josiah Trowbridge and Dr. Walter Cary. He became president of the society in 1863, and attained fame as a surgeon. He died in 1877.

Gorham F. Pratt was another physician who left the stamp of a strong individuality on the place and period of his activity. He was born at Reading, Mass., in 1804, and came to Buffalo at the age of 26 years, that is, in 1830. He entered the office of Dr. Cyrenius Chapin as a medical student and took his doctorate degree at Fairfield, N. Y., in 1831. Soon afterward he formed a partnership with Dr. Chapin, his preceptor, which continued until the death of the latter in 1838. Dr. Pratt became a member of the society in 1833, was secretary from 1834 to 1840, was elected vice-president in 1840 and president in 1841. He acquired a large practice among Buffalo's best families and was one of the most distinguished physicians of his time. He made a model secretary, as indicated by the records during the period of his service as such. He died April 5, 1871.

Lucian W. Caryl and Orson S. St. John also became members of the society in 1830. Dr. Caryl was chosen treasurer in 1834.

Dr. St. John was a native of Buffalo, where he received his preliminary education. He was educated in law at Cleveland and Cincinnati, O., and graduated in medicine at Philadelphia. His mother's house was one of two left standing when Buffalo was burned in 1813. Possessed of independent means, he practised little in either profession, but for half a century devoted himself to literature and scientific investigation, the latter especially directed toward the origin of life and celestial mechanics and resulting in the discovery of many now well-known principles. Dr. St. John was a deep student, an extensive traveler and was widely known in collegiate and scientific circles in Europe and America. After the death of his wife several years ago his home, when in this country, had been with his daughter, Mrs. Andrews, of New York. He died July 9, 1897, aged 87 years.

Horace B. Camp was vice-president in 1838 and 1841, and during the latter year he was chairman of a committee to which was referred the petition from the Medical Society of the County of Monroe, asking the coöperation of this society in procuring a repeal of the law of 1836, which obliged persons with foreign diplomas to be examined by the censors of the state society. His committee made an adverse report to the repeal of the law, but recommended such a modification of it as was proposed in 1837—namely, to the effect that physicians possessing foreign diplomas should be granted the privilege of an examination by the censors of county medical societies, or by the censors of the senatorial district in which they may reside.

James E. Hawley was elected vice-president in 1836 and president in 1837; he became a permanent member of the state medical society in 1848.

Josiah Barnes, a native of Connecticut and graduate of Yale College, took his medical degree at Jefferson in Philadelphia. He came to Buffalo in 1832 and joined the society the following year. He acted as librarian during the years 1835, 1836 and 1837; was secretary in 1840–41; president in 1842 and treasurer from 1847 to 1851 inclusive. He was one of the ablest physicians of Buffalo, a permanent member of the state society and died June 1, 1871, lamented by all who knew him.

Henceforth in the pages devoted to the consideration of this society, for the sake of convenience, a chronological record of the officers and members will be made, first giving the year, then the names of the members who joined, and, finally, the officers for the year in question. Brief sketches of prominent members who joined in each year will also be given. This will make the record easy of reference.

1834—During this year Francis L. Harris, James P. White, H. N. Munson, L. B. Benedict and Silas Smith became members.

James Platt White (1811-1881), a native of Livingston county, N. Y., commenced the study of medicine in the office of Dr. Josiah Trowbridge in 1830. This was the beginning of a medical career destined to attain the largest fame, though it was little foreseen at the time mentioned. Probably no man of his time contributed more to the history of medicine in Erie county than Dr. White. He took his doctorate degree in March, 1834, at Jefferson Medical College, Philadelphia, and in June of the same year joined the society. Dr. White was librarian in 1840; secretary during the years 1842, '43, '44 and was elected president in 1855. He represented the society in 1850 as a delegate to the state medical society and to the American medical association. He became a permanent member of the state society in 1854 and its president in 1870. In 1877 he was elected vice-president of the American medical association. In 1878, at the Buffalo meeting, he was supported for the presidency by the New

York delegation. Through the machinations of two or three designing men who shall be nameless—one yet living—he was defeated, the nominating committee standing fourteen for Dr. White and fifteen for his competitor, Dr. Theophilus Parvin. It is to the credit of the latter that he took no part in the tactics that defeated Dr. White; indeed it is probable that he was ignorant of the whole affair until after the election was over.

This is not the place in which to write an eulogium of Dr. White—that has been properly done elsewhere[1]—but it may be justly affirmed that since his decease, September 28, 1881, his place has never been filled. He was a man of affairs as well as eminent in his profession, and his relations to many enterprises looking to the prosperity of Buffalo testify to his public-spirited progressiveness. Dr. White's part in history will be referred to again when medical colleges, medical journals and hospitals are dealt with.

JAMES PLATT WHITE, M. D.

Francis L. Harris became a member of the board of health of Buffalo in 1831; health physician in 1838; vice-president of the society in 1845; president in 1846; delegate to the state society in 1836 and 1846, and a permanent member thereof in 1857.

Officers for 1834—President, Carlos Emmons; vice-president, Henry R. Stagg; secretary, Gorham F. Pratt; treasurer, Lucian W. Caryl; librarian, Erastus Burwell; censors, Josiah Trowbridge, Moses Bristol and Charles Winne.

1. Dr. Austin Flint, Trans. Med. Society State of New York, 1882, p. 337.

1835—Henry L. Benjamin, Benjamin A. Batty, H. H. Hubbard, W. H. Turner, Marcius Simons, W. H. Christison and C. H. Raymond.

It is recorded that Dr. Carlos Emmons, president of the society in 1834, was fined ten dollars for failing to deliver the president's annual address in accordance with an existing by-law.

Dr. Charles H. Raymond during the year read before the society a thesis on the use of the stethoscope, an instrument then coming into use. He acted as librarian during the years 1838, '39, '41 and '42, and was a censor for many years, but ceased to be a member in 1844.

Officers for 1835—President, Alden S. Sprague; vice-president, W. H. Pratt; secretary, Gorham F. Pratt; treasurer, Lucian W. Caryl; librarian, Josiah Barnes; board of censors, Erastus Wallis, R. Smith, Charles Winne, Bryant Burwell and Josiah Trowbridge.

1836—George Lathrop, Nelson D. Sweetland, Abraham Miller, Samuel Salisbury, Jr., William A. Greene and Brock McVickar.

Officers for 1836—President, H. H. Bissell; vice-president, J. E. Hawley; secretary, G. F. Pratt; treasurer, Charles Winne; librarian, Josiah Barnes; censors, Bryant Burwell, F. L. Harris, H. B. Camp, Jonathan Hoyt and Charles Winne. Delegate to state medical society, F. L. Harris.

1837—Franklin Fitts, Charles A. Hyde, Horatio N. Loomis, Benjamin B. Coit, Samuel M. Crawford, Nelson Peck, Jesse Merritt, Edwin Griffin and Samuel M. Abbott.

Horatio N. Loomis, a native of Connecticut, came to Buffalo in 1830, and in 1837 joined the society. He served as treasurer from 1839 to 1846 inclusive; was elected vice-president in 1851, and was sent as a delegate to the State society in 1848. During the organisation period of Buffalo Medical College Dr. Loomis had ambitions for the chair of obstetrics. It is thought by many that he never forgave his successful rival, Dr. White, who obtained and held the chair for thirty-six years. Be that as it may, there was never afterward a cordial feeling between these two men, both successful practisers of the science and art of medicine. Dr. Loomis acquired a large following, and died March 22, 1881, respected by a great community.

Samuel M. Abbott was a student of Dr. John E. Marshall and a licentiate of the Medical Society of the County of Erie. He obtained membership in 1837, which continued until 1843.

Officers for 1837—President, James E. Hawley; vice-president, Orlando Wakelee; secretary, Gorham F. Pratt; treasurer, Charles Winne; librarian,

Josiah Barnes; censors, Charles Winne, Moses Bristol, C. H. Raymond, Bryant Burwell and Carlos Emmons.

1838— —— Ford,[1] Morgan G. Lewis, Silas James, Jabez Allen, Noah H. Warriner.

Morgan G. Lewis was born in Buffalo January 15, 1813, and located at Black Rock after graduating in medicine. In 1836 he was invited to assume the duties of editor of the *Black Rock Advocate.* He became a member of the county society in 1838, and continued in that relation until his death, February 8, 1858. Dr. Lewis was a man of courteous manners and a physician of distinction.

Officers for 1838—President, Moses Bristol; vice-president, H. B. Camp; secretary, Gorham F. Pratt; treasurer, Charles Winne; librarian, C. H. Raymond; censors, Horatio N. Loomis, Josiah Barnes, Brock McVickar, Erastus Wallis and Carlos Emmons; delegate to the state society, Alden S. Sprague.

1839—Grove C. Gage, Joseph Wilder, James M. Hoyt, James Ives, J. C. Bronk.

Officers for 1839—President, Josiah Trowbridge; vice-president, Erastus Wallis; secretary, Gorham F. Pratt; treasurer, Horatio N. Loomis; librarian, C. H. Raymond; censors, Horatio N. Loomis, James P. White, Josiah Barnes, Carlos Emmons, Moses Bristol.

1840—J. B. Pride, Edmund Brown, George H. Lapham.

J. B. Pride, of Alden, was elected a member in 1840, vice-president in 1842, president in 1843. In 1849 he was appointed keeper and physician of the almshouse and was reappointed in 1850.

George H. Lapham, of Aurora, became a student in the office of Dr. Jonathan Hoyt, at Hamburg, in 1841, served for several years as a curator in the Buffalo medical college, enjoyed a large practice for many years and died December 14, 1885, aged 72 years, respected and lamented by a large community.

Officers for 1840—President, Erastus Wallis; vice-president, Gorham F. Pratt; secretary, Josiah Barnes; treasurer, Horatio N. Loomis; librarian, James P. White; censors, Elliott Burwell, Alden S. Sprague, C. H. Raymond, F. L. Harris, H. B. Camp.

1841—Austin Flint, William Van Pelt, Edwin M. Colburn, George W. Force, Nathan Way and John G. House.

Austin Flint, a native of Massachusetts, came to Buffalo in 1836 and joined the society in 1841. He was appointed health physician of Buffalo in 1842 and in 1845 established the BUFFALO MEDICAL JOURNAL. In January, 1858, he was elected president of the society, but an appointment at the New Orleans School of Medicine took him hither, hence he was not present at the annual meeting in 1859. At the June meeting, 1861, however, his presidential address was read

1. Christian name does not appear on the records.

by Dr. Sandford Eastman, the subject being, My retrospections of medical practice in Buffalo. This paper, full of interesting material, was published in the MEDICAL JOURNAL, then conducted by Dr. Miner, and is the first article in No. 1 of the new series, August, 1861. Dr. Flint died at New York, March 13, 1886, aged 73 years. In the sections on medical colleges and medical journals Dr. Flint's part in the county medical history is further considered.

AUSTIN FLINT, M. D.

William Van Pelt, who became a member in 1841, resided at Williamsville, and was president of the society in 1856; a delegate to the State society in 1859, and permanent member of the latter in 1871. Dr. Van Pelt was a man of accomplishments and enjoyed the respect and confidence of a large community. He contributed an article to the BUFFALO MEDICAL JOURNAL in 1846 entitled Epidemic erysipelas at Williamsville, one in 1855 on Epithelial cancer, and later one on Pneumonia. He acquired a large practice and died October 12, 1890, aged 75 years.

John G. House resided at Springville and was elected president in 1854. He, too, was a man of literary accomplishments and contributed an article to the BUFFALO MEDICAL JOURNAL on Erysipelas in 1846, one in 1851 entitled Remarks on the third stage of labor, and, in 1854, still another entitled Carcinoma uteri with pregnancy.

Officers for 1841—President, Gorham F. Pratt; vice-president, H. B. Camp; secretary, Josiah Barnes; treasurer, Horatio N. Loomis; librarian, C. H. Raymond; censors, Josiah Trowbridge, Charles Winne, J. B. Pride, Elliot Burwell, C. H. Raymond; delegate to state medical society, Carlos Emmons.

1842—Timothy T. Lockwood, John Mitchell, Sylvester F. Mixer, Jesse F. Locke.

Timothy T. Lockwood became a pupil of Dr. James P. White in 1834. He graduated in medicine at Philadelphia and began practice at White's Corners, remaining there ten years. Afterward he came to Buffalo and was elected mayor in 1858, serving two years. He was a man of energy and forcefulness of character. He died December 22, 1870.

Sylvester F. Mixer was born at Hornellsville, N. Y., December 15, 1815, graduated from Yale college in 1841 and took his doctorate degree from the College of Physicians and Surgeons at New York in 1847. He was appointed health physician of Buffalo in 1850 and elected president of the society in 1852. He represented the society at different times as a delegate to both the state and national bodies, becoming a permanent member of each. From 1858 to 1874 he was attending physician at the Buffalo general hospital. He was a successful and highly respected physician. He died September 16, 1883, lamented by a large circle of friends and patients.

Officers for 1842—President, Josiah Barnes; vice-president, J. B. Pride; secretary, James P. White; treasurer, Horatio N. Loomis; librarian, C. H. Raymond; censors, Carlos Emmons, Bryant Burwell, Erastus Wallis, H. H. Bissell and F. L. Harris.

1843—William K. Scott, Silas Hubbard, Horace M. Congar and Charles H. Wilcox.

The year 1843 seems to have been prolific in supplying good material to the society. William K. Scott was the first physician licensed to practise medicine by the medical society of the state of New York. His diploma was dated 1809. He came to this city from Troy, joined the medical society in 1843 and was elected president in 1844. He was a man of great energy, sterling worth and possessed a diversity of accomplishments. He lived to advanced age, became totally blind and died January 8, 1879.

Silas Hubbard also joined the society in 1843, retaining membership therein until 1855. He was a contributor to the BUFFALO MEDICAL JOURNAL and member of the Buffalo medical association, of which he was vice-president in 1851-52.

In this year, too, Horace M. Congar became a member. He was sent as delegate to the state medical society in 1854 and was elected a permanent member thereof in 1859. He was appointed by the state society as a member of a committee from the eighth senatorial district on the subject of epidemics. In 1848 he opened a private medical school for the instruction of students. He continued an active member of the society until 1875. He died a little later at an advanced age.

Charles H. Wilcox became a member in 1843 and was president in 1850. Dr. Wilcox was a decided acquisition to the society. He was an amiable and able man as well as a skilful physician. He was chosen treasurer in 1856 and again in 1857. Dr. Wilcox was the first medical officer from Buffalo to be commissioned during the war of the rebellion, and a record of his military service will be found under its appropriate head. His death, which occurred November 6, 1862, was a sad blow to the profession and the community. He will be long remembered for his sterling worth, integrity of character, and accomplishments as a physician.

Officers for 1843—President, J. B. Pride; vice-president, Jonathan Hoyt; secretary, James P. White; treasurer, Horatio N. Loomis; librarian, Josiah Trowbridge; censors, F. L. Harris, H. H. Bissell, George H. Lapham, C. H. Raymond, M. G. Lewis; delegate to state medical society, F. L. Harris.

(*Continued next month.*)

## Figures and Tables

**Figure f1:**
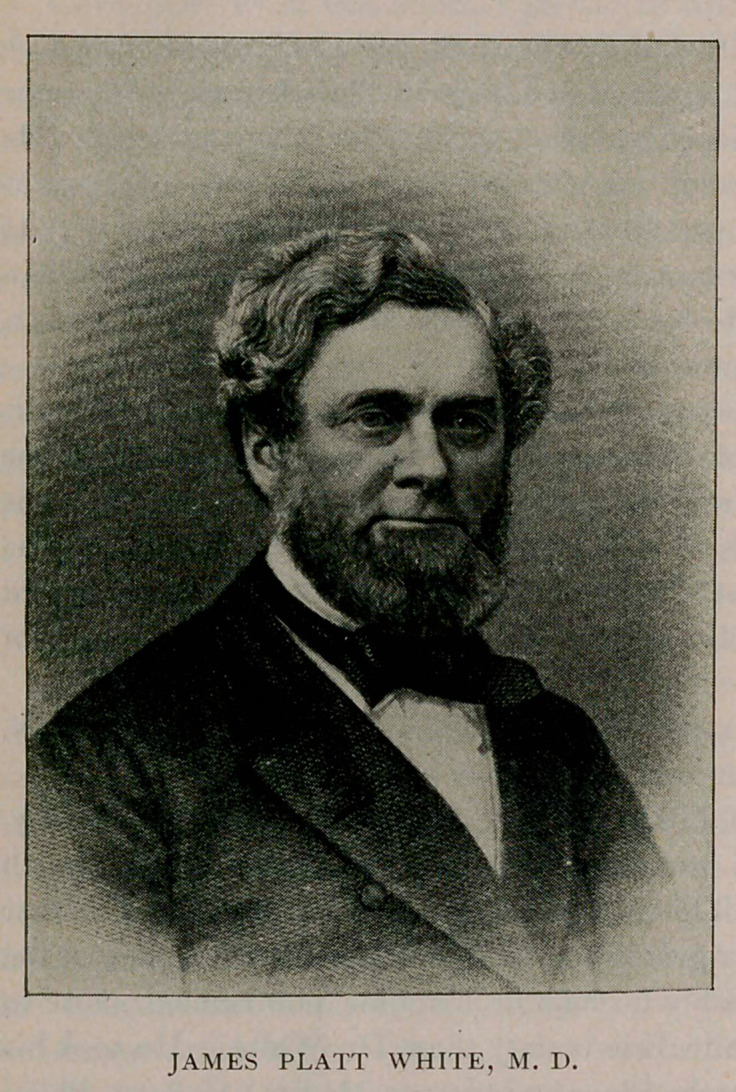


**Figure f2:**